# Ground Reaction Force Differences between Bionic Shoes and Neutral Running Shoes in Recreational Male Runners before and after a 5 km Run

**DOI:** 10.3390/ijerph18189787

**Published:** 2021-09-17

**Authors:** Xinyan Jiang, Huiyu Zhou, Wenjing Quan, Qiuli Hu, Julien S. Baker, Yaodong Gu

**Affiliations:** 1 Faculty of Sports Science, Ningbo University, Ningbo 315211, China; jiangxinyan168@163.com (X.J.); zhouhuiyu@aliyun.com (H.Z.); nbuquanwenjing@gmail.com (W.Q.); 2 School of Health and Life Sciences, University of the West of Scotland, Scotland G72 0LH, UK; 3 Savaria Institute of Technology, Eötvös Loránd University, 9700 Szombathely, Hungary; 4 Centre for Health and Exercise Science Research, Department of Sport, Physical Education and Health Hong Kong Baptist University, Hong Kong 999077, China; jsbaker@hkbu.edu.hk

**Keywords:** bionic science, ground reaction force, prolonged running, footwear

## Abstract

Running-related injuries are common among runners. Recent studies in footwear have shown that designs of shoes can potentially affect sports performance and risk of injury. Bionic shoes combine the functions of barefoot running and foot protection and incorporate traditional unstable structures based on bionic science. The purpose of this study was to investigate ground reaction force (GRF) differences for a 5 km run and how bionic shoes affect GRFs. Sixteen male recreational runners volunteered to participate in this study and finished two 5 km running sessions (a neutral shoe session and a bionic shoe session). Two-way repeated-measures ANOVAs were performed to determine the differences in GRFs. In the analysis of the footwear conditions of runners, bionic shoes showed significant decreases in vertical impulse, peak propulsive force, propulsive impulse, and contact time, while the braking impulse and vertical instantaneous loading rate (VILR) increased significantly compared to the neutral shoes. Main effects for a 5 km run were also observed at vertical GRFs and anterior–posterior GRFs. The increases of peak vertical impact force, vertical average loading rate (VALR), VILR, peak braking force and braking impulse were observed in post-5 km running trials and a reduction in peak propulsive force and propulsive impulse. The interaction effects existed in VILR and contact time. The results suggest that bionic shoes may benefit runners with decreasing injury risk during running. The findings of the present study may help to understand the effects of footwear design during prolonged running, thereby providing valuable information for reducing the risk of running injuries.

## 1. Introduction

Running has many benefits, including providing an accessible form of exercise for the cardiovascular system [1]. However, running-related injuries are common among runners, with up to 79% of runners suffering from musculoskeletal injuries annually with many factors playing a contributory role in the etiology of the injuries observed [2,3]. The high rate of running-related injuries has led healthcare providers and researchers to further understand the determinants of these injuries, as well as methods for treatment and prevention [4,5].

The majority of injuries result from overuse injuries [6]. Overuse injuries such as stress fractures, iliotibial band syndrome, patellofemoral pain syndrome, medial tibial stress syndrome and plantar fasciitis are common among recreational and competitive runners [7,8]. While the etiology of these injuries is multifactorial, landing has proven to be the most injurious [9]. Ground reaction forces (GRFs) are included in the biomechanical factors that have been often investigated during running. GRFs have been implicated as a potential cause of several running-related injuries, which relate to the body’s movement status, tissue stresses and limb-loading rates [3,10]. In a preliminary prospective study, Davis et al. [11] noticed that runners with tibial stress fractures increased lower extremity loading. Several studies [5,12,13] have reported that injured runners show higher loading rates (LR) than runners without injuries. Increased GRFs are one of the major risk factors for overuse injuries and the ability of the body to constantly absorb higher GRFs over a long period may explain the high incidence of overuse injuries in the lower extremities [14,15]. Over the last four decades, research conducted on GRFs during running has studied how these forces affect the human body and how factors such as running style, footwear design, and types of surfaces may affect GRFs [16,17,18]. To facilitate analysis, GRFs can be decomposed into three orthogonal components. The vertical ground reaction force of running has been extensively studied in the past few decades [8,11,12,18]. However, the influence of running factors on the horizontal (anterior–posterior) and sagittal (medial–lateral) components are not well established or understood.

Researchers suggested that footwear may be a risk component for running-related injuries [19]. Certain studies demonstrated that the type of footwear used has an impact on altering the GRF properties, and the relationship between the mechanical properties of footwear soles and the study of GRFs has attracted great attention in the literature [18,20,21,22]. The purpose of such studies is to design a shoe that can provide comfort, enhance running performance and reduce injuries. In recent years, footwear with an unstable sole structure has become increasingly popular due to the function of treatment [23] and functional aid [24]. Unstable sole construction may enhance neuromuscular control ability and increase muscular strength by decreasing body stability [25]. Several studies have investigated the influences of unstable sole construction. Taniguchi et al. [26] concluded that Masai barefoot technology (MBT) shoes could help to enhance shock absorption in the initial stance phase, as well as maintain the progression force and decrease joint torque and power during walking. Boyer et al. [27] found the peak first medial GRF and the peak anterior GRF decreased in the MBT shoes during running and suggested that the unstable sole structure may offer a potential treatment opportunity for running-related injuries. Sobhani et al. [28] concluded that the rocker shoes can reduce ankle plantarflexion moment among healthy individuals during running and walking. However, Nigg et al. [29] found that after training with MBT shoes for six weeks, participants have no significant enhancement in balance capacity.

Barefoot (BF) performance is an unstable condition, and barefoot running has recently become increasingly popular among runners with the opinion that barefoot running may cause fewer running-related injuries [30]. However, BF running may cause specific injuries to novice runners. Divert et al. [31] proposed that the primary aim of the shoe is to protect the human foot. In this study, bionic shoes (BS) combine the function of barefoot motion and provides protective elements. The shoes also combine traditionally unstable structures and bionic science and customize the sole according to the morphology of each participant’s foot. BS may reflect and restore barefoot movement patterns to a greater extent than other unstable shoes. Zhou et al. [32] found that participants wearing BS have bigger knee and hip flexion than wearing neutral shoes during single-leg landing, which suggested that BS might be considered to reduce the risk of lower limb injuries. In one of our previous studies [25], we investigated the effect of prolonged running with BS on the lower limb joint biomechanics and concluded that footwear personalization can bring benefits to runners, reduce the risk of injury, and improve running performance.

To the best of our knowledge, research related to the changes in the GRFs because of BS is limited, especially during a prolonged running period. Therefore, the main purpose of the present study was to compare how GRFs are changed while running using BS and typical neutral shoes (NS) before and after a 5 km running session. The results may provide useful information for future studies and shoe design relate to injury risks and performance benefits. We hypothesized that (1) most of the parameters of GRF will be decreased while running with BS either before 5 km or after 5 km running session, and (2) compared to the running test before 5 km, GRFs will be increased after the 5 km running session for both sets of shoe conditions.

## 2. Materials and Methods

### 2.1. Participants

Sixteen male recreational heel strike runners (age: 24.2 ± 1.7 years, height: 1.76 ± 0.04 m, body weight: 72.0 ± 4.6 kg, BMI: 22.8 ± 0.7 kg/m^2^) with a minimum of 20 km weekly running volume were recruited to this study. All participants had not run with bionic shoes before the experiment. Only runners who were injury-free for six months and free from health problems were included. All participants signed the documented consent form outlining the experimental design accepted by the Ethical Institutional Review Board of Ningbo University before they participated in the study.

### 2.2. Shoe Conditions

**Figure 1:**
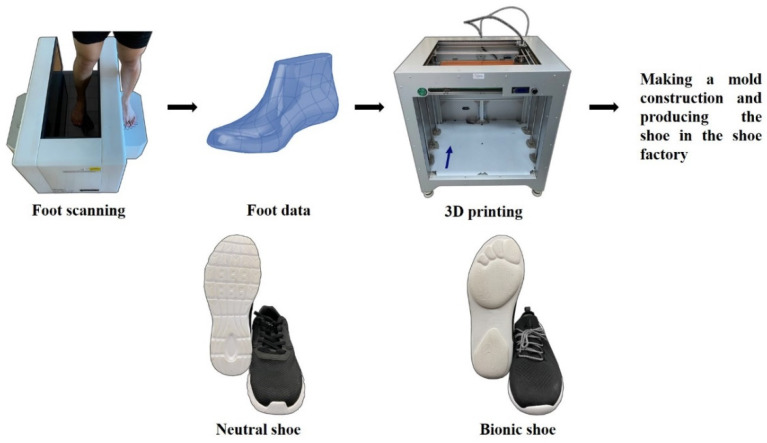
Illustration of bionic shoe-making procedure and neutral shoes [25].

Bionic shoes are characterized by the foot shape sole according to each participant’s foot, the design process consists of two parts. At first, a foot-scanning machine (VAS 39, Ortho baltic, LITHUANIA) was applied to measure individual foot shape of participants, and the second phase included using a 3D printer (Dragon(L) 3D Printer, Winbo, Guangzhou, China) to print the plastic foot model according to the scanned foot profile. After collecting individual foot information, a shoe factory (Ningbo Jiangbei Feibu Sports Goods Co., Ltd., Ningbo, China) developed the specific shoes (bionic shoes) based on the results. The structure of the sole was based on the individual shape of the foot. The control shoes were typical neutral running shoes (ART NO.11725599-7, ANTA) (Figure 1) [25]. The sole materials of both bionic shoes and neutral shoes were made of ethylene vinyl acetate (EVA) foams. More details about the shoes are given in Table 1.

### 2.3. Experimental Protocol

Participants completed two running testing sessions separately, with 7 to 10 days intervals between two sessions. In one running testing session, runners wore bionic shoes (BS). And in the other session, they wore neutral shoes (NS). The order in which participants wear shoes in the experiment was randomly selected. The experimental procedures were kept the same for two testing sessions.

A force plate (AMTI, Watertown, MA, USA) was used for collecting the GRFs at a frequency of 1000 Hz, and the force plate was mounted in the middle of an overground runway track. Participants were dressed in tight shorts and a tight-fitting short shirt. The “natural running pace” was defined as individual runners’ self-selected speed (3.09 ± 0.16 m/s, in the range of 10–12 km/h). This natural running pace was applied for all running tests (before and after the 5 km run). Participants’ running speed was evaluated and controlled by timing gates on the runway. Before the actual tests started, runners were allowed 10 min to familiarize themselves with the experimental procedures and warm up. After that, all runners finished 5 successful running trials using their dominant leg. The dominant leg was defined as the leg which the runners prefer to use when kicking a ball [25]. Successful running trials were considered as the foot of the dominant leg making contact on the force plate completely.

After the baseline running test (pre-5 km running), participants began to run 5 km on a motorized treadmill at their natural running pace. 2 min were given to runners to warm up on the treadmill, then ran at a natural running pace for 5 km. After participants completed the 5 km treadmill run, they were immediately running back to the overground runway to collect post-5 km running data, and the test protocol was the same as the pre-5 km running test.

### 2.4. Data Analysis

**Figure 2:**
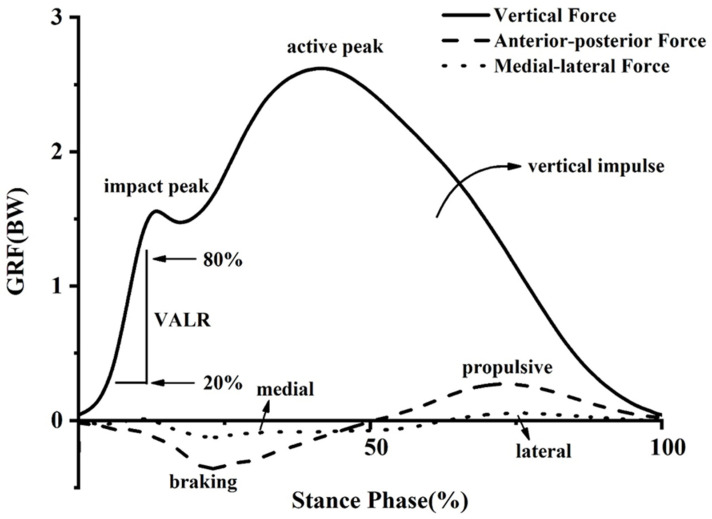
Example of ground reaction force trajectories for the stance phase of heel strike runners. Key variables of these trajectories are identified.

The ground reaction forces (GRFs) were processed with a fourth-order low-pass Butterworth filter. A threshold of 20 N on the vertical GRF was applied to identify the initial foot contact and toe-off. GRFs were subsequently normalized to the body weight (BW) of each participant. The interest GRFs were peak vertical impact force and peak vertical active force, vertical average loading rate (VALR) and maximum instantaneous loading rate (VILR), vertical impulse, contact time, peak medial force and peak lateral force, peak propulsive force and propulsive impulse, peak braking force and braking impulse (Figure 2). The braking and propulsion phases in the anterior–posterior direction were represented by negative and positive values, respectively. In the medial–lateral direction, the lateral force was considered as a positive value and the medial force was negative. These variables were the most relevant parameters selected according to previous research on GRFs during running.

The peak vertical impact force was defined as the first peak in the vertical GRF, while the peak vertical active force was defined as the second peak. The VALR and VILR were computed from 20% to 80% of the stance period between the initial foot contact and the peak vertical impact. VALR was the average slope in the period, while VILR was the steepest slope [33]. Vertical impulse was determined as the time integral of the vertical GRF over stance. Impulses were calculated as the zone surrounded by the zero lines and GRF curves for each direction [30]. Contact time was considered as the time during which a vertical force greater than 20 N was applied to the force plate.

### 2.5. Statistical Analysis

All measured parameters are presented as mean ± standard deviation. Two-way repeated-measures ANOVAs were performed to determine the main effects of ‘shoe conditions’ and ‘5 km run’ and the interaction effects of these factors on VALR, VILR, vertical impulse, contact time, peak medial force and peak lateral force, peak propulsive force and propulsive impulse, peak braking force and braking impulse. Before statistical analysis, the examination of homogeneity of residuals and normality were conducted. The alpha level was set at 0.05. When significant interaction effects were found, post hoc pairwise comparisons with the Bonferroni correction (α = p/6 = 0.008) were applied. The statistical procedures were performed using SPSS 25.0 (IBM, Armonk, NY, USA).

Since GRFs have the characteristic of one-dimensional time-varying, two-way repeated-measures ANOVAs were performed by using a one-dimensional statistical parametric mapping procedure to analyze the main effects of ‘shoe conditions’ and ‘5 km run’ and the interaction effects of two factors. The statistical analyses were finished in Matlab 2019b (The Math Works, Natick, MA, USA). The significance level was set at *p* < 0.05.

## 3. Results

### 3.1. Effects of the Shoe Conditions

**Figure 3:**
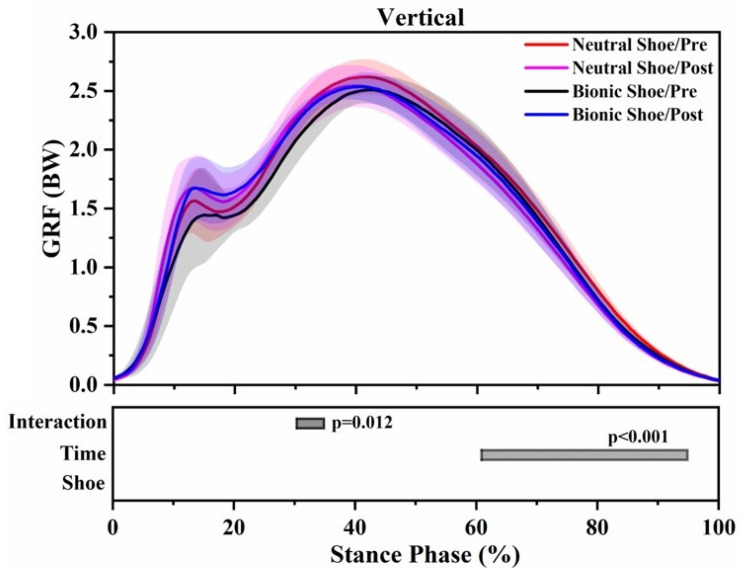

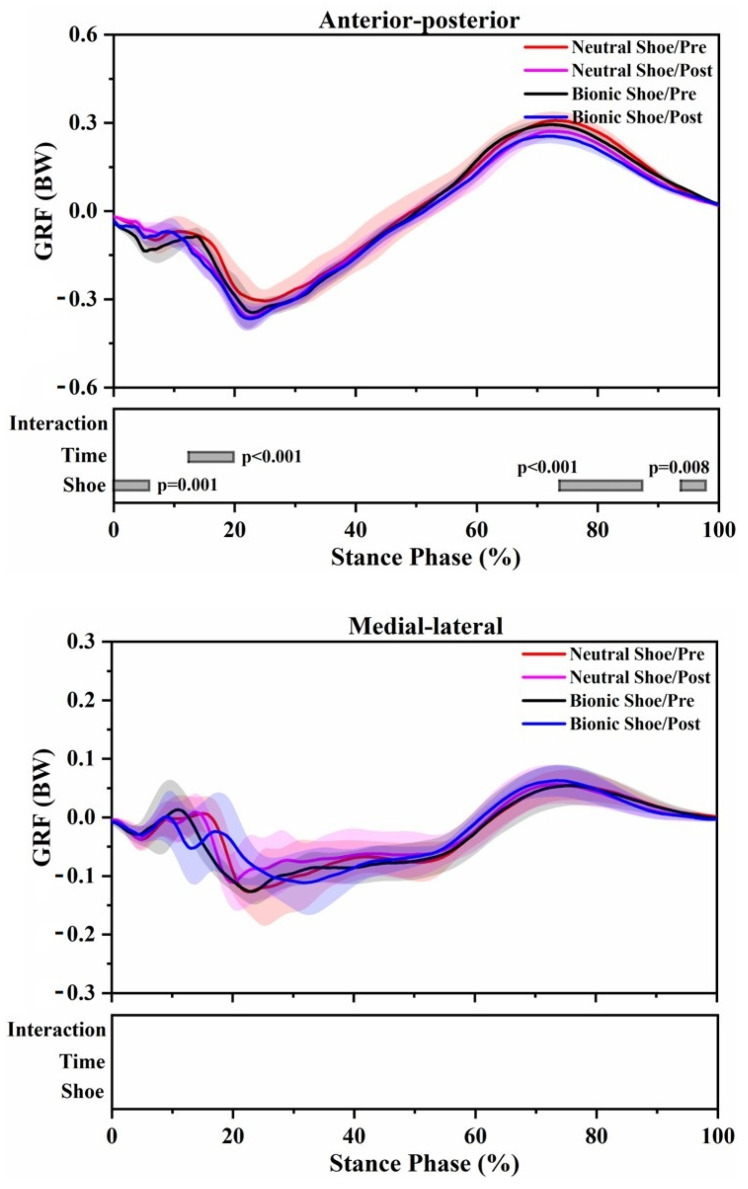
Ground reaction forces (GRFs) waveforms of the mean (SD) over the stance period of four running tests. Significant main effects of the shoe conditions, 5 km run, and interaction effects (*p* < 0.05) are highlighted (grey horizontal bars at the bottom of the figure) during consistent periods from SPM1d analyses.

In both pre-5 km running and post-5 km running, bionic shoes showed significant decreases in vertical impulse (F = 29.987; *p* < 0.001), peak propulsive force (F = 11.795; *p* = 0.004), propulsive impulse (F = 4.603; *p* = 0.049) and contact time (F = 24.714; *p* < 0.001), while the braking impulse (F = 5.273; *p* = 0.036) and VILR (F = 12.428; *p* = 0.003) increased significantly compared to neutral shoes (Table 2). In anterior–posterior GRF, the braking force of bionic shoes increased by 0–6% (*p* = 0.001), the propulsive force decreased by 74–87% (*p* < 0.001) and 94–98% (*p* = 0.008) (Figure 3). No significant differences were observed in vertical GRF and medial–lateral GRF (Figure 3).

### 3.2. Effects of the 5 km Run

For both shoe conditions, the running session after 5 km run induced significant increases in peak vertical impact force (F = 11.502; *p* = 0.004), VALR (F = 10.228; *p* = 0.006), VILR (F = 15.435; *p* = 0.001), peak braking force (F = 11.402; *p* = 0.004) and braking impulse (F = 39.430; *p* < 0.001), while significant decreases were observed in peak propulsive force (F = 21.073; *p* < 0.001) and propulsive impulse (F = 18.644; *p* = 0.001) compared to the baseline running session (Table 2). In vertical GRF, the post-5 km running decreased by 61–95% (*p* < 0.001) (Figure 3). In anterior–posterior GRF, the braking force of the post-5 km running increased by 12–20% (*p* < 0.001) (Figure 3).

### 3.3. Interaction Effects

The interaction between the shoe condition and 5 km run induced a significant effect at VILR (F = 12.729 *p* = 0.003), contact time (F = 17.551; *p* = 0.001) (Table 2) and 30–35% (*p* = 0.012) in vertical GRF (Figure 3).

## 4. Discussion

The primary purpose of the present study was to determine the effects of bionic shoes on GRFs before and after a 5 km treadmill run compared with typical neutral running shoes. The main results were that bionic shoes induced significant modifications on the GRFs during both running before or after a 5 km run, that is, bionic shoes decreased vertical impulse, peak propulsive force, propulsive impulse and contact time, while increased braking impulse and VILR and changed anterior–posterior GRF curve. We also observed that several GRFs were significantly influenced by a 5 km run, and these results support our hypothesis.

Running shoes are considered an essential part of running due to their capability to control or decrease the impact forces. When this attenuation is not well regulated, there is a risk of developing running-related injuries because of the high incidence of chronic injury in prolonged running [34]. Many researchers [4,5,13] have determined that runners with higher loading rates are at a greater risk of injury and the reduction of loading rate may be an effective way to reduce the development of injury. Our study showed a decrease in VILRs with the bionic footwear before a 5 km run, which may indicate bionic shoes have a more cushioning effect or absorb more shock during a short-term running session. In the sagittal plane of movement, vertical and horizontal components of the resultant GRF, and the corresponding impulses are the main factors that determine the running motion and the displacement of the center of mass [35]. The force impulse is related to the accumulation of creep damage or the change of the cumulative load with time or distance [36]. We observed participants regulate running state by decreasing propulsive components, vertical impulse and increasing the braking impulse during a bionic shoe running session. Since impulses are the measurement of the cumulative force over the running stance period, it is natural that there would be a resulting reduction in vertical, propulsive impulses for the bionic shoes running session, as bionic shoes imitate the state of barefoot running [30]. The braking impulse generated by the body increased during the first half of the stance and may be a strategy to maintain a steady state. As the running speed remained constant during the measurements of the two shoes, the anterior–posterior force was expected to change to compensate for posture control. Since long ground contact times may bring a high metabolic cost, due to the slow generation of force [37]. The contact time using bionic shoes was shorter than neutral shoes, and it has been implied that the capability to produce average vertical GRF in a short stance time is an advantage [38]. However, changes in the medial–lateral GRFs were not statistically significant. Alterations in lateral force may lead to the pronation of the foot, and excessive pronation has been related to lower leg and knee pain [39]. The limited studies [40,41] on medial–lateral GRF in barefoot running indicate that there are no differences in peak medial and lateral GRFs between shod and barefoot runners. The findings of our study imply that there is evidence that bionic shoes may reduce the risk of running injuries, as well as protecting the foot during running.

Long-term and high-intensity running unavoidably induces neuromuscular fatigue, which in turn affects the musculoskeletal system of the lower extremities. Generally, the motion control of the lower extremities decreases after fatigue, which is a major factor for running-related injuries [42,43]. It was expected that fatigue would influence the GRFs by altering the running state of the runners during a prolonged running session. Consistent with this assumption, most of the GRFs of the post-5 km running exhibited significant differences compared to pre-5 km running. In our study, the peak vertical impact force, VALR and VILR significantly increased, which was consistent with the findings of previous studies [44,45,46]. Studies have revealed that high vertical GRFs may increase the risks of injury for runners [13,15]. Higher impact force and loading rates may indicate that running under a fatigued state absorbs less shock, which makes the lower limbs more likely to be injured. However, the vertical GRF decreased in the late stance phase after a 5 km run. Peak braking force and braking impulse were significantly greater in post-5 km running compared to pre-5 km running, whereas peak propulsive force and propulsive impulse were smaller. The increased braking parameters may be linked to the larger vertical impact force and loading rate [18]. A previous study [47] indicated the propulsion impulse must increase in proportion to the braking impulse to provide a steady state during running, which the decreased peak propulsive force and propulsive impulse may suggest fatigue was responsible for the unstable state of running. The prolonged running session had no significant effect on medial–lateral GRFs. The three components of GRF (vertical, anterior–posterior and medial–lateral) have been independently evaluated, and there is evidence that lower vertical impact, lower peak medial–lateral force, lower braking force, and higher propulsion force are economical [37]. The results of the present study indicate that fatigue not only increases the risk of running injuries, but also reduces the economics of running.

The designs of the shoe can potentially affect sports performance and risk of injury, and different types of shoe outsoles can affect the interaction between the footwear and ground surface [48,49]. Modern running shoes are created to make rearfoot strike runners comfortable to run and less injurious by absorbing part of the transient force and spread the impulse over time [9]. The efficiency of shock absorption mechanisms is restricted by biomechanical or morphological constraints, and it is not easy to change them to improve cushioning during running. Therefore, a further improvement of the global attenuation should be applied by investigating shoe designs [22]. Generally, the GRFs of the foot strike were observed in walking and running analysis since the contact phase and its peak impact force information could potentially provide insights related to motion patterns and injury occurrence [15,48]. By contrast, certain researchers consider GRFs to be unimportant parts from an injury perspective [50,51,52]. This lack of consensus raises questions about the importance of GRFs from an injury perspective. The human body must cope with the increased external forces. Many studies have shown that larger GRFs most likely play a role in running-related injuries development, regardless of specific mechanisms [18]. There are many situations when GRFs are helpful, such as calculating inverse dynamics. More broadly, GRFs remain one of the most essential parts of sports biomechanics [36]. In the present study, the changes in GRFs have shown when running using bionic footwear compared to neutral running shoes before and after a 5 km run suggest a potential decrease in the risk of injury, as well as the risk of injury associated with fatigue. Future studies should find a compromise between running performance optimization and the protection of runners using individual shoe designs.

Our study used 3D designs to produce a specific shoe sole model system, combining solid and surface 3D models to modify the sole in real time, which also take ergonomics into consideration, for creating a new form of sports shoes. The personalized foot structure is used as the outsole for the bionic shoes, which can restore the barefoot state of human beings to the greatest extent and improve the comfort of the foot to a certain extent [53]. This may give us the inspiration that workers can wear bionic shoes in the workplace to improve comfort. However, this study was limited in ways. First, muscle activities were not measured, as muscle activities would not be the same across shoe conditions and fatigue states. Second, the shoe properties (e.g., sole hardness, heel height, and weight) of bionic shoes are not the same as neutral shoes, the differences may lead to additional interference during running. However, in addition to the sole design, the other characteristics of the two shoes are not different. The research focus of this experiment is the influence of the special bionic sole design on running biomechanics. In future research, we will study the impact of other characteristics on running biomechanics on the basis of bionic shoes. Third, bionic shoes were novel to the participants, and we did not evaluate GRFs differences after a longer period of familiarizing runners with the shoes. Allowing participants to adapt to bionic shoes over longer periods may produce different results. Future studies should investigate how runners adapt to running in bionic shoes over a period such as 4 weeks.

## 5. Conclusions

In summary, this study compared and analyzed running GRFs using bionic shoes and neutral running shoes before and after a 5 km run. Based on the results of this study, bionic shoes and a 5 km run altered ground reaction forces during running, especially the vertical and anterior–posterior forces. These findings provide preliminary evidence suggesting that bionic shoes combined with the functions of barefoot running and protective elements, traditional unstable structures and bionic science may benefit runners in reducing injury risk during running. The findings of this study may help understand the influence of footwear design and prolonged running, thereby providing useful information for running-related injuries prevention. Subsequent studies are required to investigate the changes in muscle activities, as well as further exploring the long-term influence and benefits of bionic shoes.

## Figures and Tables

**Table 1:** Information about characteristics and materials of the tested shoes.

	Neutral Shoes (NS)	Bionic Shoes (BS)
Heel height (mm)	27.0 (1.0)	23.0 (1.0)
Mass (g)	294.5 (2.3)	271.0 (2.0)
Sole hardness (Asker C)	49.6 (0.6)	50.0 (0.9)
Bending stiffness (N/mm)	13.6 (0.4)	14.2 (0.5)
Shoe upper material	nylon (polyamide) polyvinyl chloride (PVC)	nylon (polyamide) polyvinyl chloride (PVC)
Shoe sole material	ethylene vinyl acetate (EVA)	ethylene vinyl acetate (EVA)

Note: shoe properties were averaged across shoe size measurement.

**Table 2:** Mean (SD) of ground reaction forces (GRFs) characteristics for the four experimental conditions.

	Neutral Shoe/Pre	Neutral Shoe/Post	Bionic Shoe/Pre	Bionic Shoe/Post	Main Effect Shoe	Main Effect Time	Interaction Effect
Peak Vertical Impact Force (BW)	1.65 (0.29)	1.79 (0.26)	1.56 (0.34)	1.74 (0.26)	F = 1.860; *p* = 0.193	**F = 11.502; *p* = 0.004**	F = 0.301; *p* = 0.591
Peak Vertical Active Force (BW)	2.64(0.15)	2.56 (0.19)	2.55 (0.12)	2.56 (0.12)	F = 2.668; *p* = 0.123	F = 1.192; *p* = 0.292	F = 2.304; *p* = 0.150
VALR (BW/s)	61.81 (11.28)	68.55 (18.75)	54.34 (16.87)	69.91 (16.16)	F = 1.802; *p* = 0.199	**F = 10.228; *p* = 0.006**	F = 3.842; *p* = 0.069
VILR (BW/s)	119.37 (26.09)	136.76 (42.06)	113.38 (44.29)	186.13 (51.53)	**F = 12.428; *p* = 0.003**	**F = 15.435; *p* = 0.001**	**F = 12.729; *p* = 0.003**
Vertical Impulse (BW% × s)	36.79 (1.21)	36.74 (2.03)	34.91 (1.96)	34.53 (1.73)	**F = 29.987; *p* < 0.001**	F = 0.411; *p* = 0.531	F = 0.468; *p* = 0.505
Peak Medial Force (BW)	−0.16 (0.05)	−0.14 (0.04)	−0.14 (0.01)	−0.14 (0.04)	F = 1.552; *p* = 0.232	F = 1.257; *p* = 0.280	F = 1.264; *p* = 0.279
Peak Lateral Force (BW)	0.07 (0.03)	0.07 (0.03)	0.07 (0.03)	0.07 (0.03)	F = 0.838; *p* = 0.374	F = 0.324; *p* = 0.578	F = 0.387; *p* = 0.543
Peak Propulsive Force (BW)	0.31 (0.03)	0.27 (0.03)	0.30 (0.02)	0.26 (0.02)	**F = 11.795; *p* = 0.004**	**F = 21.073; *p* < 0.001**	F = 0.042; *p* = 0.840
Peak Braking Force (BW)	−0.35 (0.03)	−0.38 (0.03)	−0.36 (0.05)	−0.38 (0.04)	F = 0.598; *p* = 0.452	**F = 11.402; *p* = 0.004**	F = 0.434; *p* = 0.052
Propulsive Impulse (BW% × s)	2.28 (0.42)	1.99 (0.18)	2.15 (0.11)	1.87 (0.18)	**F = 4.603; *p* = 0.049**	**F = 18.644; *p* = 0.001**	F = 0.004; *p* = 0.951
Braking Impulse (BW% × s)	−1.95 (0.38)	−2.31 (0.32)	−2.20 (0.26)	−2.37 (0.18)	**F = 5.273; *p* = 0.036**	**F = 39.430; *p* < 0.001**	F = 2.130; *p* = 0.165
Contact Time (s)	0.26 (0.17)	0.26 (0.02)	0.25 (0.02)	0.25 (0.02)	**F = 24.714; *p* < 0.001**	F = 0.066; *p* = 0.801	**F = 17.551; *p* = 0.001**

Note: Statistical significance was set to *p* < 0.05. The significant differences in interaction effect are based on the results of Bonferroni post hoc tests (α = 0.008). The bold represents significant differences.

## Data Availability

The data that support the findings of this study are available on reasonable request from the corresponding author. The data are not publicly available due to privacy or ethical restrictions.
